# Expression profile and prognostic values of GATA family members in kidney renal clear cell carcinoma

**DOI:** 10.18632/aging.204607

**Published:** 2023-03-23

**Authors:** Xuejie Yang, Cheng Mei, Hui Nie, Jianhua Zhou, Chunlin Ou, Xiaoyun He

**Affiliations:** 1 Department of Pathology, Xiangya Hospital, Central South University, Changsha 410008, Hunan, China; 2 Department of Blood Transfusion, Xiangya Hospital, Clinical Transfusion Research Center, Central South University, Changsha 410008, Hunan, China; 3 Departments of Ultrasound Imaging, Xiangya Hospital, Central South University, Changsha 410008, Hunan, China; 4 National Clinical Research Center for Geriatric Disorders, Xiangya Hospital, Central South University, Changsha 410008, Hunan, China

**Keywords:** GATA family, kidney renal clear cell carcinoma, prognosis, methylation, immune cells

## Abstract

To investigate the possible diagnostic and prognostic biomarkers of kidney renal clear cell carcinoma (KIRC), an integrated study of accumulated data was conducted to obtain more reliable information and more feasible measures. Using the Tumor Immune Estimation Resource (TIMER), University of Alabama at Birmingham Cancer Data Analysis Portal (UALCAN), Human Protein Atlas (HPA), Kaplan-Meier plotter database, Gene Expression Profiling Interactive Analysis (GEPIA2) database, cBioPortal, and Metascape, we analyzed the expression profiles and prognoses of six members of the GATA family in patients with KIRC. Compared to normal samples, KIRC samples showed significantly lower GATA2/3/6 mRNA and protein expression levels. KIRC's pathological grades, clinical stages, and lymph node metastases were closely related to GATA2 and GATA5 levels. Patients with KIRC and high GATA2 and GATA5 expression had better overall survival (OS) and recurrence-free survival (RFS), while those with higher expression of GATA3/4/6 had worse outcomes. The role and underlying mechanisms of the GATA family in cell cycle, cell proliferation, metabolic processes, and other aspects were evaluated based on Kyoto Encyclopedia of Genes and Genomes (KEGG) and Gene Ontology (GO) enrichment analyses. Furthermore, we found that infiltrating immune cells were highly correlated with GATA expression profiles. These results showed that GATA family members may serve as prognostic biomarkers and therapeutic targets for KIRC.

## INTRODUCTION

Renal cell carcinoma (RCC) is a heterogeneous tumor originating from kidney tubular epithelial cells. RCC is one of the most widely recognized malignant tumors worldwide, representing 2.2% of all new disease cases with an estimated 431,288 new cases overall in 2020 [1, 2]. The incidence and death rate of kidney cancer are gradually rising, particularly in kidney renal clear cell carcinoma (KIRC), which comprises 75% of all kidney malignancies [3]. However, due to a lack of early identification and predictive indicators, individuals with KIRC typically have a poor prognosis [4]. KIRC is yet to be molecularly characterized. Therefore, searching for novel diagnostic or prognostic biomarkers and potential therapeutic targets [5, 6] will improve the early screening, diagnosis, and therapy of KIRC.

It has been determined that the GATA family of zinc finger DNA-binding proteins is crucial for epithelial growth and the formation of a variety of tissues [7]. Previously defined as hematological (GATA1/2/3) and cardiac (GATA4/5/6) GATA family members based on investigations of their expression, their functions and expression patterns have been shown to be widespread beyond these organs [8–10]. GATA1 and GATA2 play pivotal roles in regulating the cell cycle and proliferation [11]. GATA4 and GATA5 transcription factors are increasingly recognized as playing a role in the carcinogenesis of human tumors of endodermal and mesodermal origin [12], while GATA6 is expressed in the immature proliferating cells in the intestinal crypts and is classified as a potential oncogene [13]. The occurrence and development of cancer is a complicated process [14, 15]. Recent studies have indicated that the GATA family plays important roles in tumorigenesis, such as in lung squamous cell carcinoma [16], urothelial carcinoma [17], ovarian carcinoma [18], breast cancer [19], and gastric carcinomas [20], and the GATA family may serve as potential new biomarkers. Although KIRC-related genes and their potential biomarkers have been mentioned in a number of publications [21, 22], the prognostic significance of the GATA family in the emergence of KIRC has not been thoroughly clarified. To improve therapeutic outcomes, we employed bioinformatic techniques and research databases to evaluate GATA expression in KIRC and investigate its prognostic relevance.

## RESULTS

### Aberrantly increased expression of GATA family members in patients with KIRC

**Figure 1:**
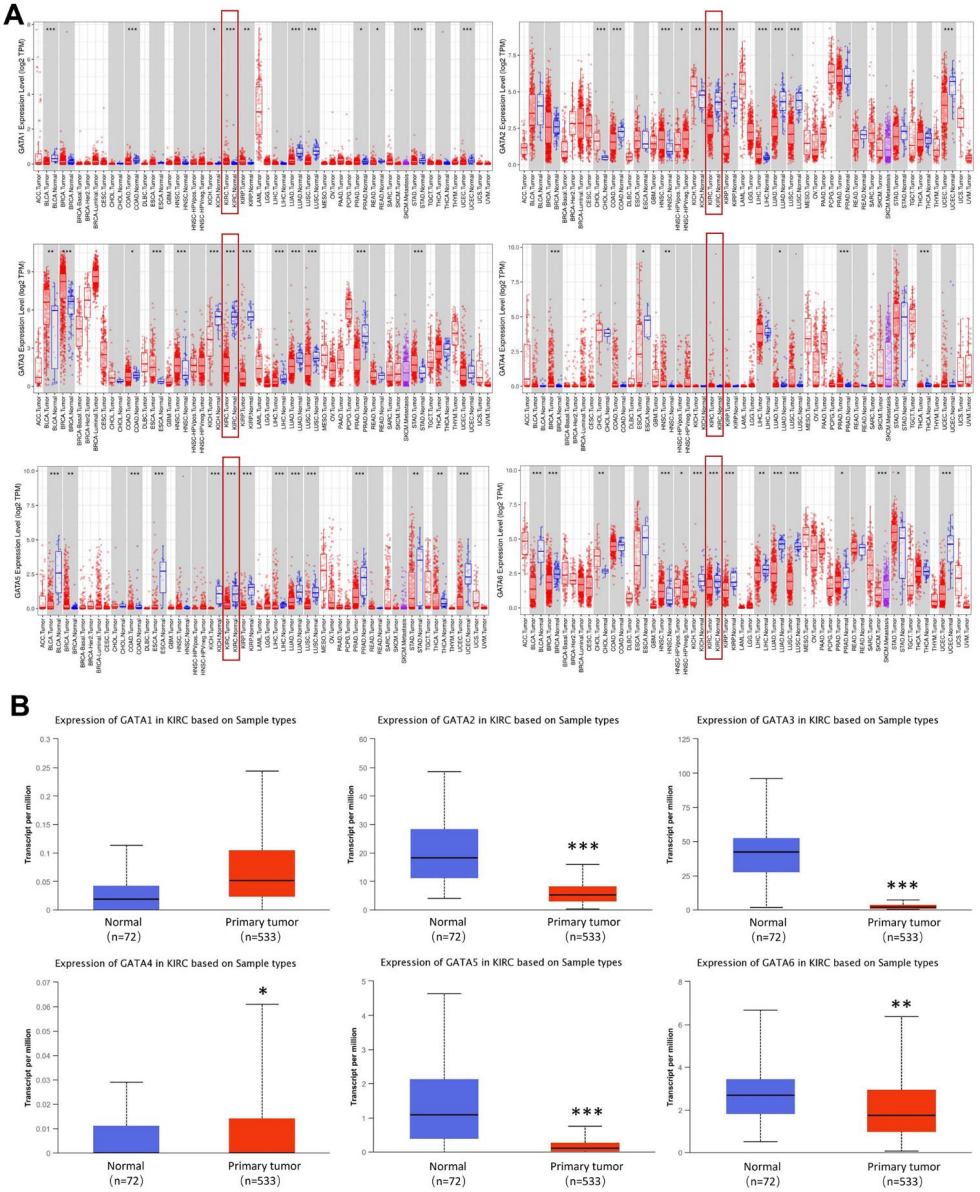
**Expression levels of GATA family members in KIRC.** (**A**) The pan-cancer expression of *GATA1-6* mRNAs. (**B**) The expression of *GATA* mRNAs in KIRC. *p < 0.05, ** *p* < 0.01, *** *p* < 0.001 compared with control. KIRC, kidney renal clear cell carcinoma.

The mRNA expression levels of GATA family members in KIRC and healthy tissues were assessed using the Tumor Immune Estimation Resource (TIMER) database. We found that *GATA2/3/5/6* expression levels were considerably downregulated in patients with KIRC. However, the expression level of *GATA1* and *GATA4* was higher in KIRC tissues than in normal tissues (Figure 1A). Then, we used the University of Alabama at Birmingham Cancer Data Analysis Portal (UALCAN) to compare the relative expression levels of GATA family members in KIRC. Notably, the results showed that the mRNA expression of *GATA2/3/5/6* was lower in KIRC tissues than in normal tissues (Figure 1B).

**Figure 2:**
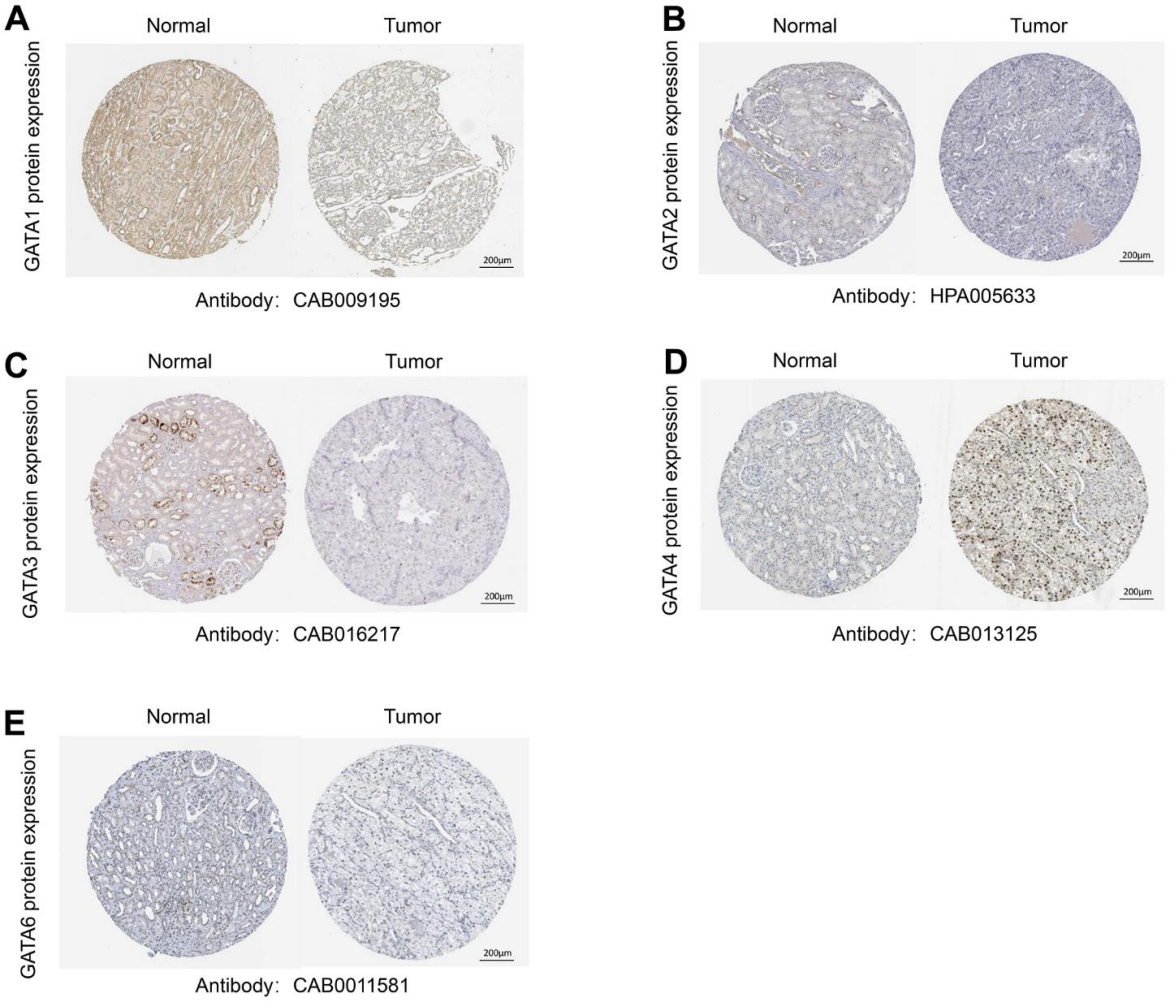
**Representative immunohistochemistry images of the GATA family members.** (**A**–**E**) The HPA database shows the protein expression levels of GATA1-6 in KIRC tissues compared with those in non-cancerous tissue. HPA, Human Protein Atlas; KIRC, kidney renal clear cell carcinoma.

We carried out immunohistochemistry analysis of the protein expression of GATA family members utilizing Human Protein Atlas (HPA) databases to further assess and validate the protein expression levels of GATA family members in KIRC. According to Figure 2, the majority of GATA family members exhibited low or no expression in KIRC tissues but moderate to high expression in normal kidney tissues. Compared to that in the corresponding normal tissues, GATA1/2/3/6 protein expression was downregulated in KIRC tissues. In contrast, GATA4 was highly expressed in KIRC (Figure 2). Moreover, the HPA database did not contain any IHC information about GATA5.

### Correlation of the expression of GATA family members with clinicopathologic features of patients with KIRC

**Figure 3:**
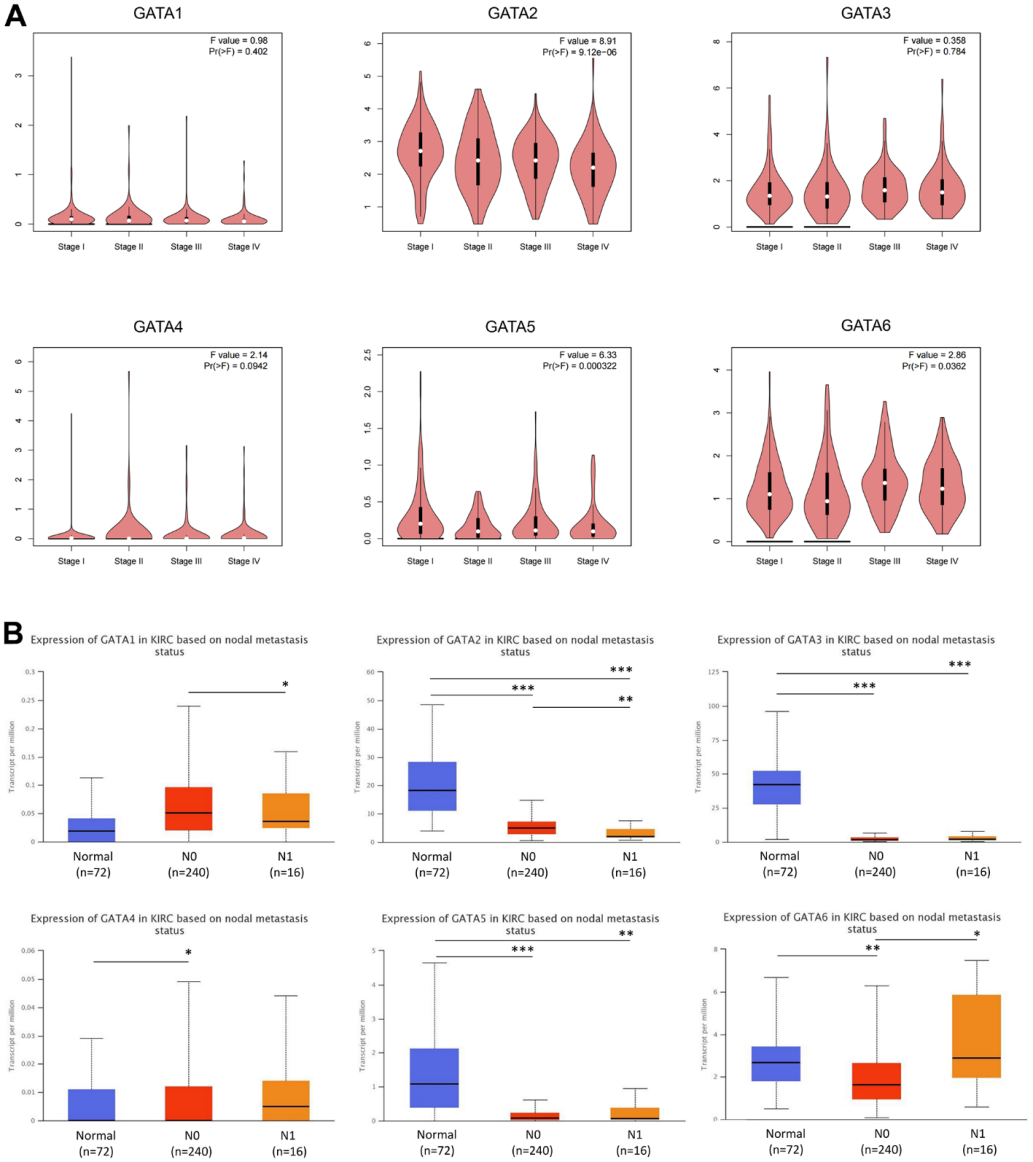
**Association of *GATA* mRNA expression levels with clinical pathology.** (**A**) The GEPIA database was used to evaluate the correlations of the expression of *GATA1-6* with the pathological stage of disease in patients with KIRC. (**B**) The relationship between mRNA expression of GATA family members and lymph node metastasis in patients with KIRC. * *p* < 0.05, ** *p* < 0.01, *** *p* < 0.001 compared with control. GEPIA, Gene Expression Profiling Interactive Analysis; KIRC, kidney renal clear cell carcinoma.

Next, the association between GATA expression and tumor stage in KIRC was investigated. GATA2/5/6 expression changed noticeably throughout the tumor stages, according to correlation analysis of TCGA data using the Gene Expression Profiling Interactive Analysis (GEPIA) database, whereas *GATA1/3/4* expression showed no discernible variations among tumor stages (Figure 3A). At the N0 and N1 phases of lymph node metastasis, we noticed that *GATA3* mRNA expression levels in KIRC tissues were lower than those in normal tissues. Additionally, the mRNA expression levels of *GATA2* and *GATA5* tended to be lower in tumors with N0 and N1 stage lymph node metastases than in normal tissues and were substantially correlated with disease prognosis, as mentioned below. Conversely, tumors with N1 stage lymph node metastases tended to have the highest level of *GATA6* mRNA expression (Figure 3B).

Moreover, we analyzed correlations between the expression of GATAs and clinicopathological characteristics using TCGA samples from patients with KIRC (Table 1). The results showed that GATA2/3/4/6 expression levels were strongly correlated with the T stage of KIRC patients. Meanwhile, GATA2/5/6 expression levels were significantly associated with the N stage of the levels of GATA2 and GATA5 were significantly associated with the M stage of KIRC patients, and the levels of GATA2 and GATA5 were significantly associated with the M stage of KIRC patients. According to these findings, members of the GATA family could be used as potential diagnostic indicators of KIRC.

**Table 1:** Clinicopathologic parameters and the expression of GATA family members in KIRC.

**Characteristics**	**N**	**GATA1**			**GATA2**			**GATA3**			**GATA4**			**GATA5**			**GATA6**		
		**Low**	**High**	***P***	**Low**	**High**	***P***	**Low**	**High**	***P***	**Low**	**High**	***P***	**Low**	**High**	***P***	**Low**	**High**	***P***
**Gender**																			
Male	337	229	108	0.071	203	134	0.416	268	69	0.127	321	16	0.689	251	86	**0.023**	217	120	0.157
Female	180	136	44		115	65		153	27		170	10		117	63		127	53	
**Age (year)**																			
≤60	258	186	72	0.457	148	110	0.053	217	41	0.118	247	11	0.427	183	74	0.900	174	84	0.664
>60	259	179	80		170	89		204	55		244	15		185	75		170	89	
**T stage**																			
T1 + T2	331	233	98	0.890	187	144	**0.002**	279	52	**0.026**	322	9	**0.001**	227	104	0.082	237	46	**0.000**
T3 + T4	186	132	54		131	55		142	44		169	17		141	45		107	127	
**N stage**																			
Nx	268	189	79	0.863	149	119	**0.011**	214	54	0.111	255	13	0.274	177	91	**0.025**	178	90	**0.041**
N0	235	167	68		158	77		198	37		224	11		181	54		161	74	
N1	14	9	5		11	3		9	5		12	2		10	4		5	9	
**M stage**																			
Mx	28	22	6	0.352	16	12	**0.001**	25	3	0.807	26	2	0.262	14	14	**0.005**	17	11	0.185
M0	412	285	127		240	172		336	76		393	19		291	121		282	130	
M1	77	58	19		62	15		60	17		72	5		63	14		45	32	
**Pathologic stage**																			
Stage I + II	313	216	97	0.326	173	140	**0.000**	266	47	**0.010**	305	8	**0.001**	215	98	0.122	225	88	**0.001**
Stage III + IV	204	149	55		145	59		155	49		186	18		153	51		119	85	
**Histologic grade**																			
grade 1 + 2	238	161	77	0.174	123	115	**0.000**	196	42	0.619	235	3	**0.000**	155	83	**0.005**	172	66	**0.011**
grade 3 + 4	279	204	75		195	84		225	54		256	23		213	66		172	107	

Bold font indicates significant difference.

### Prognostic value of the GATA family in patients with KIRC

**Figure 4:**
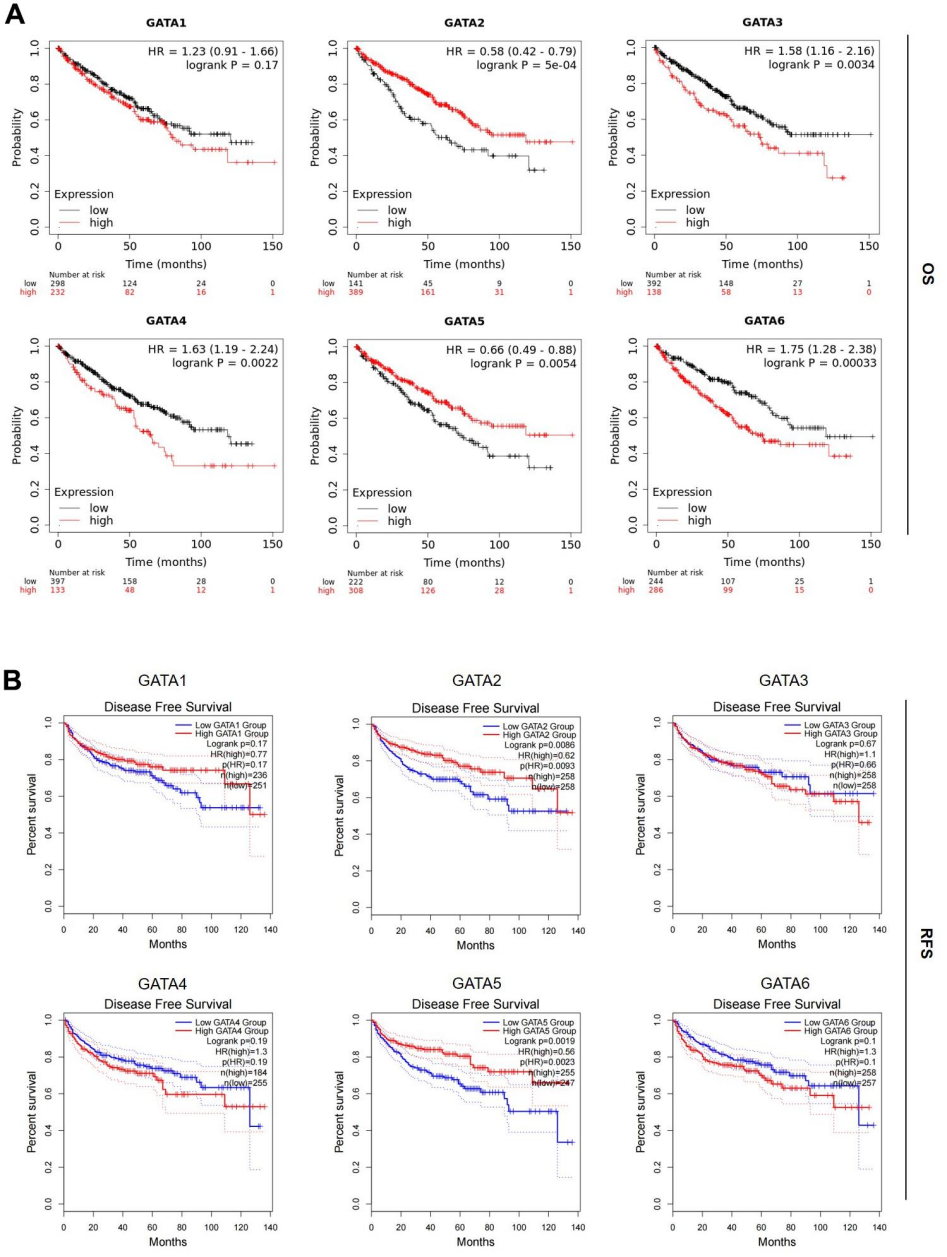
**Prognostic value of the mRNA expression levels of the *GATA* family members in patients with KIRC.** (**A**, **B**) The OS and RFS of *GATA1–6* in patients with KIRC were analyzed by the Kaplan–Meier plotter and GEPIA2, respectively. GEPIA2, Gene Expression Profiling Interactive Analysis 2; KIRC, kidney renal clear cell carcinoma; OS, overall survival; RFS, recurrence-free survival.

Next, we examined the prognostic significance of *GATA* mRNA expression in patients with KIRC, including overall survival (OS) and recurrence-free survival (RFS), using the Kaplan–Meier plotter and the GEPIA2 databases. In patients with KIRC, we discovered that higher *GATA2* and *GATA5* expression levels were substantially correlated with longer OS and higher *GATA3/4/6* expression levels were associated with poorer prognosis (Figure 4A). Similarly, we found that in individuals with KIRC, higher *GATA2* and *GATA5* expression was substantially linked with better RFS (Figure 4B). Other GATA factor mRNA expression levels showed no appreciable impact on OS and RFS in patients with KIRC. According to our findings, increased *GATA2* and *GATA5* expression was substantially linked with prolonged OS and RFS in patients with KIRC, suggesting that GATA2 and GATA5 are potential biomarkers for the prognosis of KIRC, with higher expression indicating better outcomes.

Using the Kaplan–Meier plotter database, we further analyzed the prognostic value of GATA family members in different clinical stages and pathological grades of KIRC (Table 2). Abundant expression of *GATA2* was significantly associated with shorter OS in stage II but was significantly correlated with better OS in stages I, III, and IV KIRC. The mRNA expression of *GATA3* was closely related to poorer OS in patients with stage III and grade III KIRC and correlated with longer OS in those with stage II KIRC. Moreover, *GATA4* transcriptional expression was significantly associated with worse OS in patients with stage III and better OS in those with grade III KIRC. We also found that high expression of *GATA1* and *GATA6* was correlated with poor OS in patients with stage II and stage IV KIRC. When considered collectively, our findings indicate that several GATA family members are potential prognostic markers in KIRC and are particularly useful for predicting the OS of patients with KIRC.

**Table 2:** Kaplan–Meier plotter was used to analyze the prognostic value of GATA family members in different pathological grades and clinical stages of KIRC.

	**GATA1**		**GATA2**		**GATA3**		**GATA4**		**GATA5**		**GATA6**	
	**HR**	***P***	**HR**	***P***	**HR**	***P***	**HR**	***P***	**HR**	***P***	**HR**	***P***
**Stage1**	1.53(0.73-3.19)	0.257	0.43(0.19-0.96)	**0.034**	1.66(0.77-3.57)	0.192	0.66(0.35-1.27)	0.210	0.56(0.-1.02)	0.055	1.79(0.92-3.48)	0.083
**Stage2**	3.89(1.19-12.75)	**0.016**	3.08(1.03-9.23)	**0.035**	0.14(0.02-1.07)	**0.027**	0.55(0.18-1.64)	0.275	2.69(0.73-9.83)	0.121	-	**0.001**
**Stage3**	0.67(0.37-1.21)	0.183	0.52(0.29-0.94)	**0.027**	2.08(1.16-3.72)	**0.012**	1.88(1.04-3.42)	**0.034**	0.68(0.39-1.2)	0.184	0.52(0.29-0.94)	**0.028**
**Stage4**	1.98(1.15-3.42)	**0.012**	0.48(0.28-0.8)	**0.005**	1.49(0.91-2.44)	0.107	1.63(0.98-2.7)	0.057	0.64(0.39-1.04)	0.072	1.89(1.01-3.55)	**0.044**
**Grade2**	1.35(0.66-2.73)	0.408	0.53(0.23-1.18)	0.114	0.53(0.25-1.1)	0.085	1.443(0.77-2.67)	0.260	0.64(0.35-1.15)	0.133	1.86(0.96-3.61)	0.063
**Grade3**	1.53(0.96-2.42)	0.071	1.33(0.84-2.1)	0.230	2.38(1.49-3.78)	**0.000**	0.56(0.35-0.91)	**0.017**	0.59(0.32-1.07)	0.081	1.68(0.98-2.86)	0.055
**Grade4**	1.56(0.89-2.73)	0.119	0.68(0.4-1.17)	0.165	1.71(0.91-3.19)	0.090	1.3(0.75-2.24)	0.353	0.62(0.35-1.11)	0.106	1.25(0.69-2.24)	0.460

Bold font indicates significant difference.

### Genetic alteration of GATA family members in patients with KIRC

**Figure 5:**
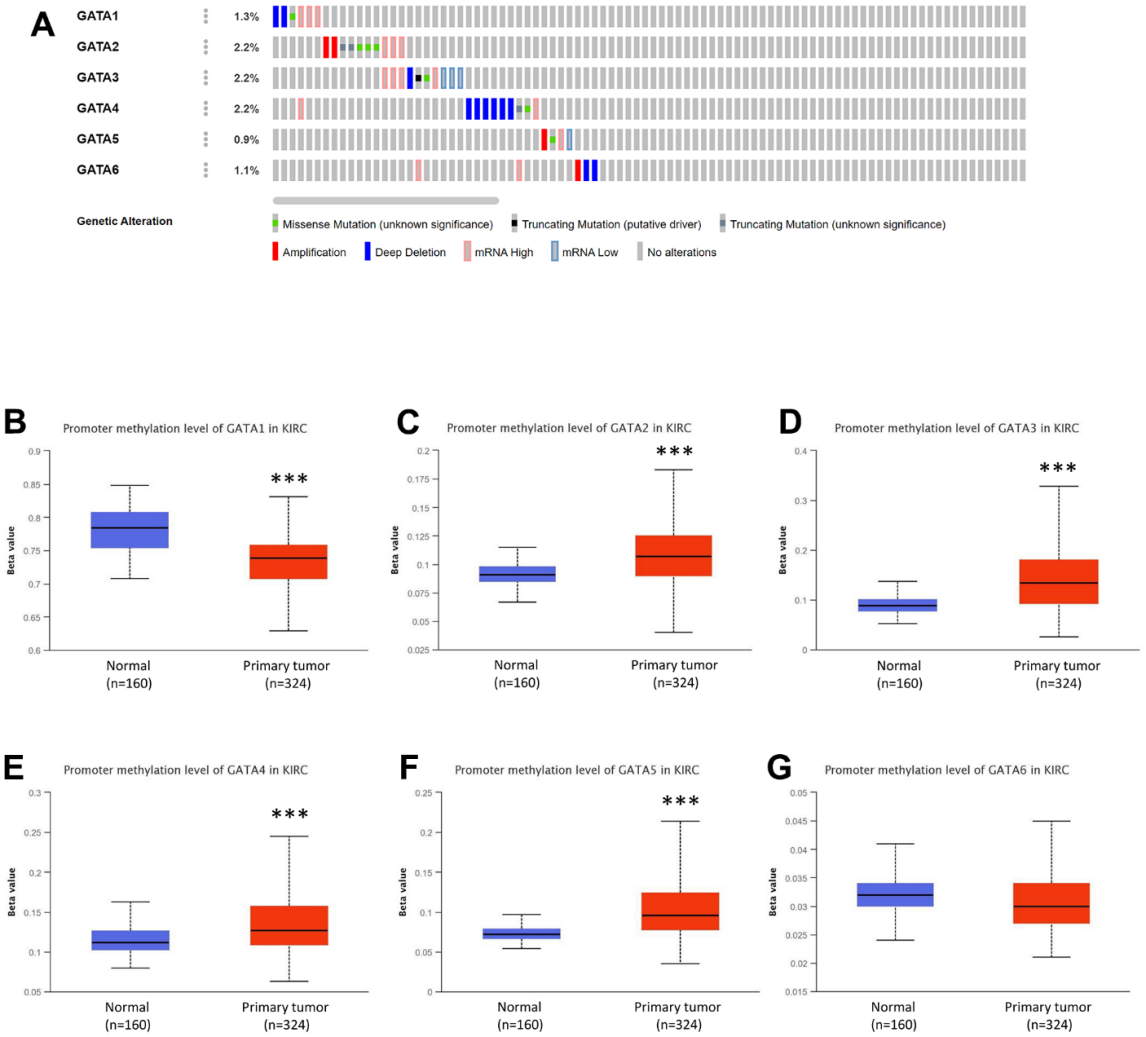
**Genetic alterations and DNA methylation levels of distinct *GATA* family members in KIRC.** (**A**) Summary of the alteration rates for *GATA1–6* in KIRC (cBioPortal). (**B**–**G**) DNA methylation changes in *GATA1-6* in KIRC assessed using the UALCAN database. *** *p* < 0.001 compared with control. KIRC, kidney renal clear cell carcinoma; UALCAN, University of Alabama at Birmingham Cancer Data Analysis Portal.

Utilizing the TCGA database and the online tool cBioPortal, the profiles of genomic changes for each GATA member are shown in Figure 5. Thirty-nine (9%) of the 446 enrolled individuals with KIRC had altered GATA family genes in total. Among the GATA family members, GATA 2/3/4 had the highest genetic alteration rate (2.2%), followed by GATA1 (1.3%), GATA6 (1.1%), and GATA5 (0.9%) (Figure 5A). mRNA high and deep deletions were the two predominant genetic alteration types in the GATA family members. Rarely did the GATAs show amplification, missense, in-frame, or splicing mutations.

The DNA methylation levels of *GATA* family members in patients with KIRC were also detected through the UALCAN database. *GATA1* had considerably lower DNA methylation levels in KIRC samples than in healthy human controls, while *GATA2/3/4/5* showed significantly higher levels in KIRC tissues and *GATA6* showed statistically negligible variations between normal and malignant tissues (Figure 5B–5G).

### Gene Ontology (GO) and Kyoto Encyclopedia of Genes and Genomes (KEGG) enrichment analysis of protein-protein interactions (PPIs) of GATAs

**Figure 6:**
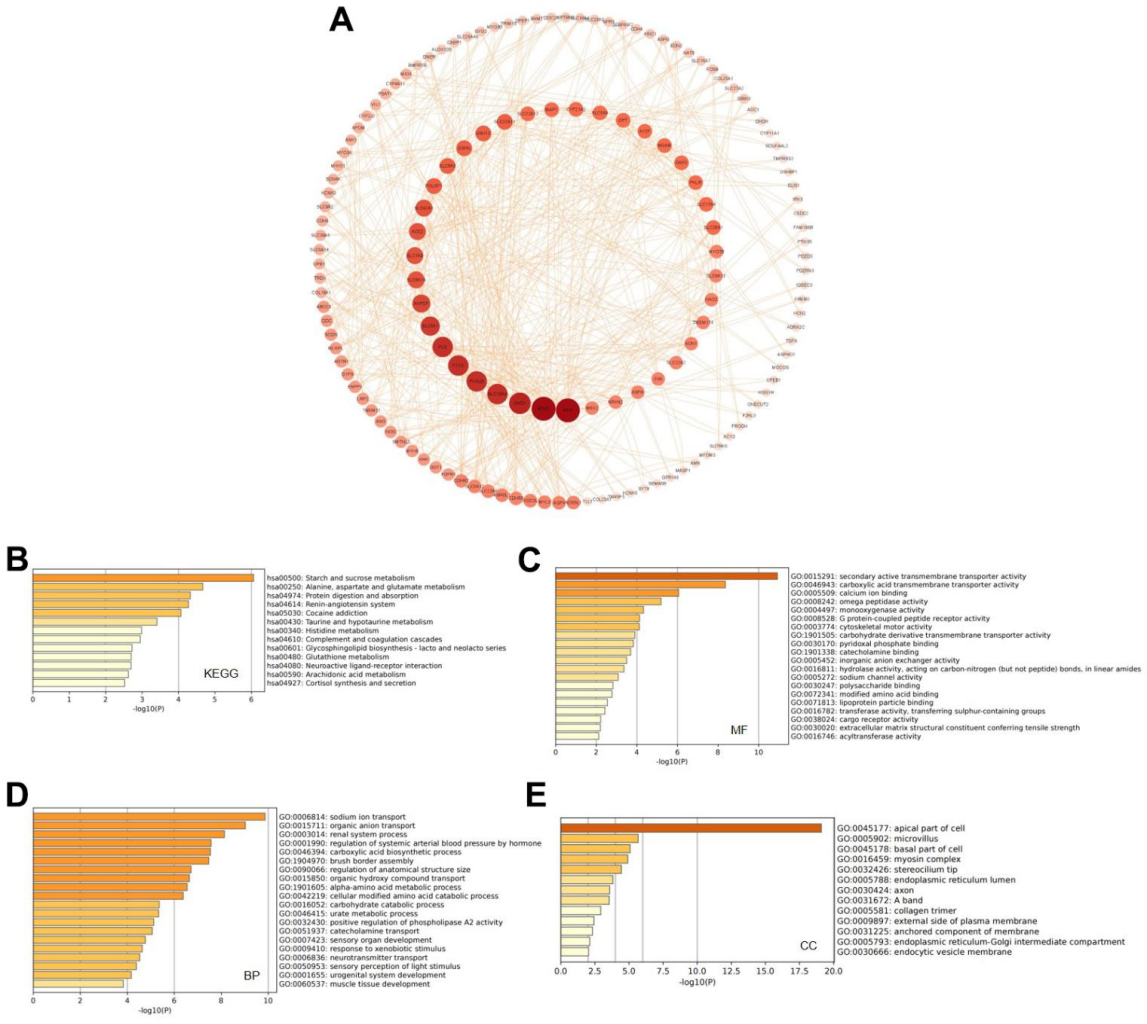
**Predicted functions and pathways of GATAs and GATA-associated co-expressed molecules in KIRC.** (**A**) 277 GATA-associated co-expressed molecules that were most frequently altered in KIRC were identified using the cBioPortal database. The PPI network was generated from the GATA family members and their associated co-expressed genes, which was constructed using the Cytoscape database. (**B**–**E**) GO functional enrichment analysis and K EGG pathway analysis of GATA-associated co-expressed molecules were conducted using the Metascape database. KIRC, kidney renal clear cell carcinoma; PPI, protein-protein interaction; GO, Gene Ontology; KEGG, Kyoto Encyclopedia of Genes and Genomes.

A PPI network was constructed based on the top 277 genes that were co-expressed and related to the GATAs, which were identified using cBioPortal and Cytoscape (Supplementary Table 1). REN, BDNF, GAD, SLC16A9, PVALB, FTCD, and PLG had the highest likelihood of interacting with the GATAs and promoting KIRC development (Figure 6A).

In addition, we used the 277 identified co-expressed genes to further analyze the potential function of GATAs in KIRC by analyzing their GO terms and KEGG pathways using the Metascape database. The KEGG results showed that the co-expressed genes were mainly related to starch and sucrose metabolism; alanine, aspartate, and glutamate metabolism; and protein digestion and absorption. Likewise, there was a connection between the GATAs and the complement and coagulation cascades (Figure 6B). The enriched GO pathways for molecular function were secondary active transmembrane transporter activity, carboxylic acid transmembrane transporter activity, calcium ion binding, and omega peptidase activity (Figure 6C). The top enriched pathways for biological process were sodium ion transport, organic anion transport, renal system processes, regulation of systemic arterial blood pressure by hormones, and carboxylic acid biosynthetic processes (Figure 6D). Cellular component analysis revealed that these genes were frequently related to the apical part of the cell, microvillus, basal part of the cell, and myosin complex (Figure 6E).

### Immune cell infiltration and GATA family expression in patients with KIRC

**Figure 7:**
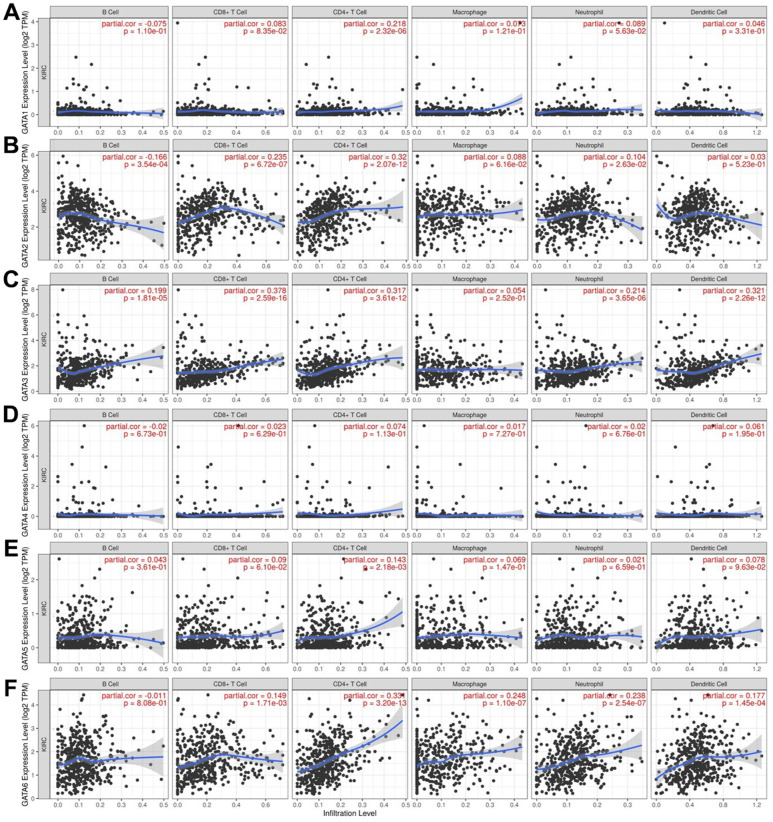
**Association of *GATA* mRNA expression levels with immune cell infiltration.** (**A**–**F**) The associations of *GATA1–6* with immune cell infiltration were evaluated using the TIMER database. TIMER, Tumor Immune Estimation Resource.

The relationship between differential GATA family expression and immune cell infiltration was analyzed using the TIMER database (Figure 7). *GATA3* and *GATA6* were both positively correlated with CD8+ T cells, CD4+ T cells, neutrophils, and dendritic cells (cor > 0.1, *p* < 0.05). In addition, *GATA3* was positively correlated with B cells, and *GATA6* was positively correlated with macrophages (cor > 0.1, *p* < 0.05). *GATA1* and *GATA5* were positively correlated with CD4+ T cells (cor > 0.1, *p* < 0.05). *GATA2* was negatively correlated with B cells (cor < -0.1, *p* < 0.05) and positively correlated with CD8+ T cells, CD4+ T cells, and neutrophils (cor > 0.1, *p* < 0.05).

In the TIMER database, we next searched for any connections between the expression of GATA family members and immunological signature markers of different immune cells infiltrating KIRC (Table 3). *GATA1* levels were associated with M1 and M2 macrophages, dendritic cells, natural killer cells (NKs), Th1, Th2, T follicular helper (Tfh) cells, Th17, and regulatory T cells (Tregs). The infiltration of CD8+ T cells, T cells, tumor-associated macrophages (TAMs), M1 macrophages, dendritic cells, NKs, Th1, Th2, Th17, and Tregs in KIRC was strongly correlated with *GATA2* expression. A notable link was observed between *GATA3* and the markers for CD8+ T cells, B cells, T cells, TAM, M1 and M2 macrophages, neutrophils, dendritic cells, NKs, Th1, Th2, Tregs, T cell exhaustion, and monocytes. *GATA4* mRNA expression was moderately or poorly correlated with M1 and M2 macrophages, neutrophils, Th1, Th17, and Treg gene markers but substantially correlated with other immune cells. *GATA5* expression showed a positive association with dendritic cells, NKs, Th1, Th2, and Th17 cells. Furthermore, we found that *GATA6* mRNA expression was closely associated with markers for CD8+ T cells, B cells, T cells, TAM, M1 and M2 macrophages, neutrophils, dendritic cells, NKs, Th1, Th2, Tfh, Th17, Tregs, T cell exhaustion, and monocytes.

**Table 3:** The correlations between the expression of GATA family members and the markers of immune cells.

		**GATA1**		**GATA2**		**GATA3**		**GATA4**		**GATA5**		**GATA6**	
		**Cor**	***P***	**Cor**	***P***	**Cor**	***P***	**Cor**	***P***	**Cor**	***P***	**Cor**	***P***
CD8+ T cell	CD8A	-0.043	0.325	-0.109	*	0.366	***	0.041	0.35	0.058	0.18	0.144	***
	CD8B	-0.042	0.333	-0.123	**	0.367	***	0.042	0.34	0.084	0.05	0.119	**
	GZMA	-0.034	0.439	-0.088	*	0.387	***	0.043	0.32	0.061	0.16	0.113	**
B cell	CD19	0.019	0.666	-0.097	*	0.322	***	0.071	0.10	0.025	0.56	0.258	***
	CD79A	0.015	0.729	-0.067	0.12	0.311	***	0.073	0.09	0.019	0.67	0.262	***
	MS4A1	0.091	*	0.056	0.20	0.336	***	0.067	0.12	0.106	*	0.297	***
T cell	CD3D	-0.017	0.695	-0.130	**	0.409	***	0.051	0.24	0.061	0.16	0.157	***
	CD3E	-0.008	0.855	-0.068	0.12	0.416	***	0.059	0.18	0.055	0.20	0.168	***
	CD2	-0.023	0.591	-0.102	*	0.413	***	0.064	0.14	0.052	0.23	0.167	***
TAM	CCL2	0.080	0.064	0.089	*	-0.005	0.90	0.016	0.72	0.185	***	0.134	**
	CD68	-0.032	0.455	-0.251	***	-0.105	*	-0.005	0.91	-0.036	0.41	0.090	*
	IL10	0.084	0.053	-0.036	0.41	0.196	***	0.094	*	0.024	0.58	0.266	***
M1	IRF5	-0.056	0.200	-0.257	***	0.019	0.66	0.052	0.23	0.073	0.09	-0.027	0.53
	PTGS2	0.148	***	0.204	***	0.096	*	0.085	*	0.011	0.80	0.317	***
	NOS2	0.339	***	0.485	***	0.039	0.37	0.046	0.28	0.426	***	0.281	***
M2	MS4A4A	0.071	0.103	0.023	0.60	0.092	*	0.070	0.11	0.004	0.93	0.309	***
	CD163	0.126	**	0.038	0.38	0.031	0.47	0.085	*	0.044	0.31	0.282	***
	VSIG4	0.041	0.344	-0.087	*	0.040	0.36	0.097	*	0.048	0.27	0.302	***
Neutrophils	ITGAM	0.053	0.225	-0.049	0.26	0.071	0.10	0.067	0.12	0.065	0.13	0.227	***
	CCR7	0.142	**	0.043	0.32	0.246	***	0.127	**	0.025	0.56	0.296	***
	SIGLEC5	0.049	0.262	0.006	0.88	0.101	*	0.000	1.00	0.106	*	0.149	***
DC	HLA-DQB1	0.074	0.086	-0.018	0.67	0.189	***	0.043	0.33	0.171	***	0.077	0.08
	HLA-DPB1	0.014	0.742	-0.050	0.25	0.218	***	0.018	0.68	0.144	***	0.090	*
	HLA-DRA	-0.019	0.663	-0.099	*	0.184	***	0.032	0.46	0.089	*	0.084	0.05
	HLA-DPA1	-0.017	0.695	-0.049	0.26	0.209	***	0.009	0.83	0.096	*	0.094	*
	ITGAX	0.034	0.427	-0.121	**	0.141	**	0.125	**	0.023	0.59	0.080	0.06
	CD1C	0.182	***	0.184	***	0.139	**	0.068	0.12	0.326	***	0.262	***
	NRP1	0.285	***	0.502	***	-0.016	0.71	-0.004	0.92	0.218	***	0.314	***
NK cell	KIR2DL1	0.173	***	0.253	***	0.002	0.96	-0.046	0.29	0.138	**	0.094	*
	KIR2DL3	0.128	**	0.170	***	0.065	0.13	-0.043	0.32	0.118	**	0.108	*
	KIR2DL4	-0.059	0.173	-0.002	0.96	0.195	***	0.056	0.20	0.024	0.58	0.087	*
	KIR3DL1	0.155	***	0.252	***	0.053	0.22	-0.017	0.69	0.152	***	0.078	0.07
	KIR3DL2	0.051	0.239	0.153	***	0.122	**	0.026	0.55	0.064	0.14	0.077	0.08
	KIR3DL3	0.045	0.298	0.054	0.21	-0.026	0.55	0.087	*	-0.051	0.24	0.043	0.32
	KIR2DS4	0.062	0.153	0.235	***	0.044	0.31	-0.049	0.26	0.103	*	0.088	*
Th1	TBX21	0.198	***	0.287	***	0.296	***	0.049	0.26	0.177	***	0.200	***
	STAT1	-0.047	0.274	-0.136	**	0.241	***	0.103	*	0.113	**	0.178	***
	STAT4	0.093	*	0.066	0.13	0.291	***	0.114	**	0.057	0.19	0.254	***
	IFNG	-0.068	0.117	-0.185	***	0.335	***	0.041	0.35	0.044	0.31	0.113	**
Th2	STAT6	0.130	**	0.267	***	-0.029	0.50	-0.023	0.60	0.174	***	0.022	0.61
	GATA3	0.008	0.845	0.160	***	1.000	***	0.072	0.09	0.130	**	0.194	***
	STAT5A	-0.017	0.689	-0.020	0.65	0.265	***	0.056	0.20	0.084	0.05	0.186	***
	IL13	0.127	**	0.088	*	0.057	0.19	0.059	0.17	0.061	0.16	0.072	0.10
Tfh	BCL6	0.166	***	0.076	0.08	0.019	0.66	0.025	0.56	0.010	0.82	0.171	***
Th17	STAT3	0.224	***	0.277	***	0.083	0.05	0.030	0.49	0.209	***	0.285	***
	IL17A	-0.005	0.902	-0.075	0.08	0.023	0.59	0.114	**	-0.055	0.21	0.068	0.12
Treg	FOXP3	-0.045	0.297	-0.202	***	0.282	***	0.111	*	-0.069	0.11	0.222	***
	STAT5B	0.256	***	0.476	***	-0.003	0.94	-0.058	0.18	0.271	***	0.104	*
	CCR8	-0.050	0.246	-0.125	**	0.232	***	0.078	0.07	-0.068	0.12	0.190	***
	TGFB1	0.177	***	0.247	***	0.162	***	0.138	**	0.002	0.97	0.380	***
T -cell exhaustion	PDCD1	-0.047	0.275	-0.188	***	0.346	***	0.046	0.29	0.054	0.22	0.100	*
	CTLA4	-0.047	0.283	-0.196	***	0.318	***	0.116	**	-0.016	0.71	0.167	***
	HAVCR2	0.003	0.952	-0.070	0.11	-0.053	0.22	0.019	0.67	0.094	*	-0.010	0.82
	LAG3	-0.066	0.129	-0.208	***	0.352	***	0.084	0.05	0.027	0.53	0.143	***
Monocyte	CD86	-0.056	0.197	-0.151	***	0.163	***	0.042	0.34	0.015	0.74	0.167	***
	C3AR1	0.036	0.402	-0.045	0.29	0.067	0.12	0.038	0.39	0.069	0.11	0.172	***
	CSF1R	0.036	0.402	0.024	0.59	0.121	**	0.070	0.11	0.086	*	0.256	***

Correlation R value was calculated by Spearman’s algorithm and adjusted by tumor purity.

*P < 0.05, **P < 0.01, ***P < 0.001.

## DISCUSSION

Key modulatory proteins known to operate as transcription factors that regulate several pathways include members of the GATA family. These GATA transcription factor-related pathways, however, are still not completely understood. As important transcription factors, the GATA family members make ideal and alluring targets to research cutting-edge treatments for KIRC. GATA2 may play a tumor suppressor role in acute myeloid leukemia (AML), and Casey et al. proposed that restoring its function (when inactivated) may be advantageous for patients with AML [23]. Feng et al. demonstrated that an increase in the expression of GATA5 inhibited the expression of β-catenin and reprogramming genes and suppressed tumor growth, colony formation, metastasis, and invasion, while promoting apoptosis in KIRC cells [24]. Wang et al. demonstrated that GATA5, as a tumor suppressor, could inhibit the progression of prostate cancer by regulating the expression of PLAGL2 [25]. In addition, GATA5 suppressed cholangiocarcinoma cell growth and metastasis via the Wnt/β-catenin pathway [26]. Numerous studies have indicated aberrant expression of members of the GATA family in diverse types of tumors, suggesting their vital roles in tumorigenesis and cancer progression [27–29]. As far as we are aware, the GATA family's function in KIRC has not been systematically examined. Thus, the mRNA expression levels of GATA family members were analyzed in KIRC tissues and their levels were compared with those in healthy kidney tissues using the TIMER and UALCAN databases. In comparison to normal tissues, KIRC tissues showed lower expression levels of GATA2/3/5/6, indicating patients with KIRC had lower levels of the GATA2/3/6 proteins.

Recent studies have indicated that GATA family members could be widely used as promising biomarkers for the clinicopathological diagnosis of various cancers. Satoshi et al. revealed that in urothelial carcinoma, GATA3 is one of the most useful markers in diagnostic surgical pathology and may serve as a reliable prognostic marker in patients with urothelial carcinoma [30]. Grainne et al. found that GATA6 regulates epithelial-mesenchymal transition and tumor dissemination and is a marker of adjuvant chemotherapy response in pancreatic ductal adenocarcinoma (PDAC) [31, 32]. Moreover, Andrés et al. demonstrated that GATA4 is a potential marker of tumor growth in PDAC and that the expression of GATA4 and GATA6 is a biomarker of poor prognosis and therapeutic response [33]. The clinical association and prognostic significance of abnormally expressed GATAs in patients with KIRC were then investigated. We discovered a connection between KIRC clinicopathological staging and GATA2/5/6 expression levels. Moreover, the data demonstrated a strong correlation between lymph node metastasis and *GATA2/3/5* mRNA expression levels in KIRC tissues. According to our research, GATA2 and GATA5 were associated with a better OS and RFS in patients with KIRC, and GATA3/4/6 overexpression was associated with a poorer prognosis. These findings indicate that these gene family members, particularly GATA2 and GATA5, have prognostic significance, great promise for patient prognosis, and tremendous potential as diagnostic markers in patients with KIRC.

The accumulation of genetic alterations and the resulting changes in gene expression patterns are regarded as the main forces behind tumor progression [34]. Patients with KIRC were discovered to have alterations in each of the six members of the GATA family, with a total genetic alteration rate of 9.9%. Additionally, DNA methylation contributes to the growth of tumors and is linked to levels of gene expression [35]. The involvement of *GATA* DNA methylation in malignancies may be deduced from a number of indicators. For instance, early gastric carcinogenesis frequently involves the epigenetic inactivation of *GATA4* and *GATA5* by CpG island methylation, which is strongly linked with *Helicobacter pylori* infection [20]. Fu et al. demonstrated that *GATA5* is rarely methylated in normal duct epithelium but is highly methylated in pancreatic cancer tissue [12]. The DNA methylation of *GATA4* and *GATA5* is a common and specific event in colorectal cancer. *In vitro* studies have shown that GATA4 and GATA5 have a tumor- inhibitory effect in colorectal cancer cells [36]. In this investigation, we demonstrated that the higher levels of DNA methylation in KIRC tissues may be the cause of the decreased expression levels of GATA2/3/5.

Then, 277 co-expressed genes and the molecular biological functions of GATA members were examined. The modulation and function of the differentially expressed GATAs in KIRC were most closely associated with REN, BDNF, GAD, and SLC16A9, according to protein-protein network interactions. KEGG pathway analysis showed that complement and coagulation cascades were specifically related to GATAs. A system of plasma proteins called the complement cascade is triggered when infections are present. Complement activation can occur through the classical, lectin, or other routes. Each of these routes produces an essential enzyme activity that further induces effector molecules of the complement system. Complement activation has three major effects: it opsonizes pathogens, recruits inflammatory and immunocompetent cells, and directly kills pathogens [37]. Our findings imply that GATA-mediated signaling, via affecting the recruitment of immunocompetent cells, may play critical roles in antitumor immunity.

Although KIRC is a well-known heterogeneous disease, useful biomarkers that contribute to individualized treatment options are still lacking, especially for current immunotherapies [38]. Recent studies have shown that GATAs may serve as therapeutic targets in cancer immunotherapy. Fu et al. identified that GATA2 drives PD-L1 and PD-L2 expression, and PD-L2 correlated with worse clinical outcomes in patients with gliomas. Targeting GATA2 may help reduce the inhibitory effects of PD-L2 in the tumor microenvironment [39]. Moreover, tumor-associated macrophages (TAMs) possess great potential in affecting the development of ovarian cancer (OC). Chen et al. found that TAM-derived extracellular vesicles allowed for the transfer of GATA3 into OC cells, which facilitated the immune escape of OC cells and their resistance to cisplatin [40]. Therefore, GATA3 might serve as a potential immunotherapeutic target for OC.

In this study, we discovered an astonishing relationship between the expression of certain GATA family members and the infiltration of six immune cell types. As a result, we investigated the link between immune infiltration markers in KIRC and the expression of GATA family members. Interestingly, a number of immune cells showed substantial associations with the expression of GATA family members. These findings imply that the immunological state of KIRC may be reflected by the expression of GATA family members, which may also serve as targets for immunotherapeutic approaches in the future. However, our study has certain limitations. For example, when we analyzed the GATA family members in KIRC, we only used a series of websites or databases; further *in vivo* and *in vitro* experiments are needed to corroborate our findings.

## CONCLUSIONS

In conclusion, using bioinformatics methods, we thoroughly examined the expression and predictive capacity of the GATA family members in patients with KIRC in an effort to further our knowledge of the critical involvement of these transcription factors in tumor development and immune responses in patients with KIRC. GATA2 and GATA5 may be novel predictive indicators and possible targets for the personalized treatment of these patients, according to our comprehensive bioinformatics investigation. However, further research is needed to assess the mechanism of their influence on tumor development/progression and identify new pharmacological therapies. The results in this study may help clarify the distinctive functions of GATAs in KIRC.

## MATERIALS AND METHODS

### TIMER database

Based on the TCGA database, the TIMER database (https://cistrome.shinyapps.io/timer/) is a comprehensive resource that can evaluate immune cell infiltration and the clinical impact of 10,897 tumors from 32 different cancer types. Numerous features of TIMER include survival analysis, gene expression comparisons between tumor and normal tissues in various malignancies, and investigation of the relationships between genes and immune-invading cells [41, 42]. The mRNA expression of GATA family members in different tumors or particular cancer subtypes from TIMER was examined in our study. Log-scale values were calculated as log2 [TPM (Transcripts per million)]. The expression of the GATA members and the infiltration of six immune cell types, including B cells, CD8 + T cells, CD4 + T cells, macrophages, neutrophils, and dendritic cells, in KIRC were also analyzed using the TIMER database.

### GEPIA2 database

The GEPIA2 database (http://gepia2.cancer-pku.cn/) is an open access dataset that offers vital interactive and programmable features, such as differential expression, pathological stage, and patient survival analyses. To assess the relationships between GATA family expression and the clinical stage and RFS of patients with KIRC, we employed the GEPIA2 database. According to the median expression of single GATA family members, the patients with KIRC were divided into low and high GATA family member expression groups.

### The HPA

The HPA (https://www.proteinatlas.org/) is an online database that contains immunohistochemistry-based expression data for various cancer types [43, 44]. In this investigation, we used immunohistochemical images to examine the levels of protein expression of several GATA members in KIRC tumors and normal kidney tissues.

### UALCAN database

The UALCAN database (http://ualcan.path.uab.edu/) is an interactive web resource based on RNA-sequence levels and clinical data from 31 cancers in the TCGA database [45]. UALCAN was applied in this study to determine the mRNA expressions of GATA family members in KIRC tissues and their correlation with nodal metastatic status. Additionally, utilizing the UALCAN database, we forecasted DNA methylation alterations in the GATA family members in KIRC tissues.

### Kaplan–Meier plotter database

To assess the relationships between GATA family expression and the OS of patients with KIRC, we employed the Kaplan–Meier plotter database (https://kmplot.com). According to the median expression of single GATA family members, the patients with KIRC were divided into low and high GATA family member expression groups. Kaplan–Meier analysis was assessed by log-rank tests, and statistical significance was considered at *p* < 0.05.

### cBioPortal

Cancer genomes and clinical data were analyzed using the cBioPortal platform (https://www.cbioportal.org/) [46]. The genomic map of the GATA family, which contains information on mutations and mRNA expression, was examined in this study. The threshold of |log2FC| was 1, and the *p*-value cutoff was 0.01.

### STRING

The STRING database investigates possible protein interaction networks. The GATA family of genes and related genes were used by STRING to construct the PPI network.

### Cytoscape

In this study, 277 co-expressed molecules of the GATA family members that were identified through cBioPortal underwent functional integration using the Cytoscape platform (Supplementary Table 1).

### Metascape

Metascape (http://metascape.org) is a complete, potent, adaptable, and interactive set of web-based analytic tools [47]. We used this platform to carry out GO and KEGG enrichment analyses.

### Statistical analysis

SPSS (version 26.0) was used to conduct statistical analysis on the relationships between the clinicopathological characteristics of patients with KIRC and the mRNA expression of GATA family members. Student's t-tests were used for comparisons and *p*-values less than 0.05 were considered statistically significant.

### Data availability statement

The original contributions presented in the study are included in the article/Supplementary Material, further inquiries can be directed to the corresponding authors.

## Supplementary Material

Supplementary Table 1
